# Health-related quality of life in patients with gastrointestinal stromal tumor: data from a real-world cohort compared with a normative population

**DOI:** 10.1016/j.esmorw.2024.100037

**Published:** 2024-04-24

**Authors:** D. van de Wal, D. den Hollander, I.M.E. Desar, H. Gelderblom, A.W. Oosten, A.K.L. Reyners, N. Steeghs, W.T.A. van der Graaf, O. Husson

**Affiliations:** 1 Department of Medical Oncology, The Netherlands Cancer Institute, Amsterdam; 2 Department of Medical Oncology, Radboud University Medical Center, Nijmegen; 3 Department of Medical Oncology, Leiden University Medical Center, Leiden; 4 Department of Medical Oncology, Erasmus MC Cancer Institute, Erasmus University Medical Center, Rotterdam; 5 Department of Medical Oncology, University Medical Center Groningen, University of Groningen, Groningen; 6 Department of Clinical Pharmacology, The Netherlands Cancer Institute, Amsterdam; 7 Department of Psychosocial Research and Epidemiology, The Netherlands Cancer Institute, Amsterdam; 8 Department of Surgical Oncology, Erasmus MC Cancer Institute, Erasmus University Medical Center, The Netherlands

**Keywords:** GIST, health-related quality of life, patient-reported outcomes, EORTC QLQ-C30, treatment setting, fatigue

## Abstract

**Background:**

Treatment and follow-up (FU) care procedures for gastrointestinal stromal tumors (GISTs) impose great challenges on patients and could potentially affect their health-related quality of life (HRQoL). The aims of our study were to (i) assess HRQoL among patients with GIST in different treatment phases and settings and to compare this with the HRQoL of an age- and sex-matched normative population, (ii) determine the occurrence of disease- and treatment-specific symptoms, and (iii) investigate which sociodemographic and clinical characteristics and symptoms were associated with HRQoL.

**Methods:**

A total of 328 Dutch patients with GIST (response rate 63%) completed a one-time survey including the European Organization for Research and Treatment of Cancer Quality of Life Core Questionnaire (EORTC QLQ-C30), which was used to assess HRQoL. HRQoL scores are presented as means and standard deviations (mean ± SD), and were compared with those of an age- and sex-matched normative population.

**Results:**

The global QoL of patients receiving imatinib in a curative setting (mean ± SD 81.2 ± 12.6) was comparable with the normative population (mean ± SD 77.1 ± 18.2), while patients who had completed their curative treatment [including those discharged from FU (mean ± SD 85.2 ± 14.0) and still in FU (mean ± SD 82.7 ± 15.0)] had a significant better global QoL with comparable functioning scores. Patients on tyrosine kinase inhibitors in a palliative setting scored significantly lower on global QoL (mean ± SD 71.6 ± 19.4) and all functioning scales compared with the normative population. HRQoL was most affected by fatigue, in addition to pain, dyspnea, and financial difficulties, which all occurred more often in patients treated in a palliative setting compared with patients in the curative setting.

**Conclusion:**

With these results, medical oncologists can reassure patients with GIST treated in an adjuvant setting that their HRQoL will not be permanently affected by imatinib and provide appropriate support to patients in the palliative setting.

## Highlights

• Patients on palliative tyrosine kinase inhibitor treatment had worse global QoL and functioning.
• Patients in a palliative setting more often suffer from symptoms that affect HRQoL.
• HRQoL was most affected by fatigue, pain, dyspnea, and financial difficulties.
• Patients with GIST on tyrosine kinase inhibitors reported lower scores on cognitive functioning.
• GIST survivors who had completed their curative treatment scored higher on global QoL than the normative population.

## Introduction

Gastrointestinal stromal tumor (GIST) is a rare cancer, affecting 10-20 individuals per million per year.^1^^,^^2^ GIST can arise anywhere in the gastrointestinal tract, predominantly in the stomach (60%) and small intestine (25%),^3^^,^^4^ with liver or peritoneal metastasis at the time of diagnosis in one in five patients.^2^ For localized GISTs, surgical resection is the mainstay of treatment, combined with (neo)adjuvant imatinib in case of large tumors, or anticipated surgery-related morbidity, or high-risk disease.^5^^,^^6^ Patients with metastatic GIST, by contrast, often depend on life-long treatment with tyrosine kinase inhibitors (TKIs), of which imatinib is the first line, and sunitinib, regorafenib, and ripretinib are registered as second-, third-, and fourth-line therapies, respectively.^5^

Before the introduction of imatinib, the median survival of patients with metastatic GIST was poor, but imatinib phenomenally improved their median survival from 12 to 68 months.^7^ TKIs, especially imatinib, are often described as tolerable, even though most patients experience side-effects.^8^ Besides side-effects, patients can face various psychosocial challenges,^9^, ^10^, ^11^ including fears, (sc)anxiety, uncertainties, and relationship and financial difficulties. Both side-effects and psychosocial challenges can hamper a patient’s health-related quality of life (HRQoL). HRQoL is a multidimensional concept that includes the patient’s perception of the impact of the disease and its treatment on physical, psychological, and social functioning.^12^ HRQoL in patients with GIST has been marginally studied, as the majority of studies have focused on objective (e.g. radiological response) and physician-reported (e.g. adverse events) outcomes,^8^ while the importance of patient-reported outcomes (PROs) in research and cancer care in general has been shown.^13^ Therefore the aims of our study were to (i) assess HRQoL among patients with GIST in different treatment phases and settings and to compare this with the HRQoL of an age- and sex-matched normative population, (ii) determine the occurrence of disease- and treatment-specific symptoms, and (iii) investigate which sociodemographic and clinical characteristics and symptoms were associated with HRQoL.

## Methods

Data from the ‘Life with GIST’ study were used, which was approved by the Medical Ethical Committee of the Radboud University Medical Center (2019-5888). According to the medical ethical regulations, the approval of one ethical committee for survey research is valid for all participating centers. The design of this study, study population, and data collection were described previously.^14^ In summary, this cross-sectional study was conducted among patients registered in the Netherlands Cancer Registry (NCR), diagnosed with GIST between 2008 and 2018, and treated within one of the five GIST reference centers. Patients were not eligible when they had a cognitive impairment or were too ill at the time of the study based on the advice of their (former) treating specialist. All patients provided written informed consent, including permission to link their study data to data from the NCR and Dutch GIST Registry (DGR). Data collection took place from September 2020 through June 2021 in the Patient-Reported Outcomes Following Initial treatment and Long-term Evaluation of Survivorship (PROFILES) registry.^15^

### Measures

Patients self-reported sociodemographic data, educational level, and comorbidities via the Self-administered Co-morbidity Questionnaire.^16^ Gender, socioeconomic status, and tumor characteristics were derived from the NCR. Socioeconomic status was based on the median household income within a postal code level. Treatment characteristics were derived from the DGR. The European Organization for Research and Treatment of Cancer Quality of Life Core Questionnaire (EORTC QLQ-C30)^17^ was used to assess HRQoL, calculating global QoL, functioning (physical, role, cognitive, emotional, and social), and symptom scale (fatigue, nausea and vomiting, pain, dyspnea, insomnia, appetite loss, constipation, diarrhea, and financial difficulties) scores.^18^ Higher scores indicate a better quality of life or functioning for the global QoL and functioning scales, and a higher symptom burden for the symptom scales. To assess disease- and treatment-specific symptoms we used the nine symptom scales of the EORTC QLQ-C30. As these nine scales do not cover (all) common symptoms and side-effects that patients with GIST may experience, we added eight items of the EORTC Symptom-Based Questionnaire,^19^ and four items of the EORTC Item Library.^20^

### Normative population

HRQoL data of an age- and sex-matched normative sample without cancer from 2017 was obtained from CentERdata, using a household panel representative of the population in the Netherlands. The panel members were randomly matched based on sex and age at the time of EORTC QLQ-C30 completion.

### Nonresponders

Nonresponder data were not shared with the research team. An anonymous comparative analysis between responders and nonresponders was conducted by an NCR employee (Supplementary Table S1, available at https://doi.org/10.1016/j.esmorw.2024.100037).

### Statistical analysis

All statistical analyses were carried out using SPSS Statistics (version 29.0’ IBM Corporation, Armonk, NY). Two-sided *P* values of <0.05 were considered statistically significant. Categorical variables were reported as numbers and percentages, and continuous variables as means and standard deviations (SDs). For our analysis, except the uni- and multiple linear regression analyses, we categorized patients into four groups: (i) completed curative treatment and discharged from follow-up (FU), (ii) completed curative treatment and still in FU, (iii) on TKI with curative intent, and (iv) on (former) TKI with palliative intent. To compare sociodemographic and clinical characteristics and HRQoL scale scores, chi-square tests and analysis of variance tests with *post hoc* Bonferroni tests were conducted. In the main text of this paper, the focus is on the main results of the comparison of HRQoL scores between patients with GIST and the normative population. In Supplementary Table S2, available at https://doi.org/10.1016/j.esmorw.2024.100037, a complete overview of the results is presented. In the case of statistical significance between groups regarding HRQoL scales, the clinical relevance of the difference in mean scores was determined according to the evidence-based guidelines.^21^ Multiple linear regression analyses were carried out to examine the association between sociodemographic and clinical variables and symptom scales of the EORTC QLQ C-30 with a *P* value of <0.1 in the univariate linear regression analysis, and the outcomes global QoL, and physical, role, cognitive, emotional, and social functioning. The included variables were checked for multicollinearity using the variance inflation factors and variance proportions test. The key results of the multiple linear regression are addressed in the main text of this paper. In Supplementary Table S3, available at https://doi.org/10.1016/j.esmorw.2024.100037, the results of the complete analysis are presented.

### Sensitivity analysis

As current TKI treatment can lead to side-effects that may overlap with the symptom scales included in the analyses, which may affect the outcomes, the linear regression analyses were repeated in the group of patients on TKI treatment. The results of our sensitivity analyses are presented in Supplementary Table S3, available at https://doi.org/10.1016/j.esmorw.2024.100037. In the main text of this paper, the differences between the multiple linear regression and the sensitivity analyses are discussed.

## Results

A total of 328 patients with GIST (response rate 63%) with a mean age of 66.7 years participated; 53% were male and two-thirds had one or more comorbidities (Table 1). Patients were on average 5.9 years after their GIST diagnosis. The majority (91.5%) received surgery for the GIST and 219 patients (66.8%) received TKI treatment at some point, of which 120 (36.6%) were on TKIs at the time of the survey, 27 with a curative and 93 with a palliative intent, respectively. Of the 328 patients, 208 (63.4%) patients completed their treatment, of which 147 were still in FU and 61 had been discharged from FU.

**Table 1:** Sociodemographic and clinical characteristics of the study population

		Completed treatment		(Current) TKI treatment			
	Total GIST sample (*n* = 328)	Not in FU (*n* = 61)	Still in FU (*n* = 147)	Curative (*n* = 27)	Palliative (*n* = 93)	Normative population (*n* = 828)	*P* value
Sex, *n* (%)							0.772
Male	174 (53.0)	34 (55.7)	72 (49.0)	15 (55.6)	53 (57.0)	439 (53.0)	
Female	154 (47.0)	27 (44.3)	75 (51.0)	12 (44.4)	40 (43.0)	389 (47.0)	
Age at survey completion, mean ± SD	66.7 ± 10.4	67.9 ± 11.6	65.9 ± 10.1	63.7 ± 11.9	68.0 ± 9.2	64.7 ± 13.9	0.057
Socioeconomic status, *n* (%)							0.322
Low	150 (45.7)	27 (44.3)	62 (42.2)	11 (40.7)	50 (53.8)		
High	178 (54.3)	34 (55.7)	85 (57.8)	16 (59.3)	43 (46.2)		
Marital stage, *n* (%)							**<0.001**^a^
Married/living with a partner	246 (75.7)	43 (70.5)	108 (75.0)	23 (85.2)	72 (77.4)	523 (63.2)	
Not living with a partner	79 (24.3)	18 (29.5)	36 (25.0)	4 (4.8)	21 (22.6)	305 (36.8)	
Missing	3	**—**	3	**—**	**—**	**—**	
Educational level^b^, *n* (%)							0.776
Low/intermediate	206 (64.0)	35 (58.3)	95 (66.4)	15 (57.7)	61 (65.6)	525 (63.4)	
High	116 (36.0)	25 (41.7)	48 (33.6)	11 (42.3)	32 (34.4)	303 (36.6)	
Missing	6	1	4	1	**—**	**—**	
Comorbidity, *n* (%)							0.359^a^
None	109 (33.4)	20 (32.8)	52 (35.6)	10 (37.0)	27 (29.3)	220 (26.6)	
1	71 (21.8)	13 (12.3)	32 (21.9)	5 (18.5)	21 (22.8)	232 (28.0)	
≥2	146 (44.8)	28 (45.9)	62 (42.5)	12 (44.4)	44 (47.8)	376 (45.4)	
Missing	2	**—**	1	**—**	1	**—**	
Type of comorbidity, *n* (%)							
Heart disease	40 (12.2)	7 (11.5)	21 (14.3)	5 (18.5)	7 (7.6)	116 (14.0)	
Stroke	5 (1.5)	2 (3.3)	1 (0.7)	**—**	2 (2.2)	11 (1.3)	
High blood pressure	95 (29.1)	19 (31.1)	48 (32.7)	4 (14.8)	24 (26.1)	239 (28.9)	
Lung disease	32 (9.8)	6 (9.8)	17 (11.6)	4 (14.8)	5 (5.4)	71 (8.6)	
Diabetes	26 (8.0)	5 (8.2)	14 (9.5)	2 (7.4)	5 (5.4)	61 (7.4)	
Stomach disease	4 (1.2)	**—**	1 (0.7)	1 (3.7)	2 (2.2)	10 (1.2)	
Kidney disease	9 (2.8)	**—**	4 (2.7)	**—**	5 (5.4)	10 (1.2)	
Liver disease	10 (3.0)	**—**	2 (1.4)	**—**	8 (8.6)	8 (1.0)	
Anemia or other blood disease	20 (6.1)	2 (3.3)	4 (2.7)	3 (11.1)	11 (12.0)	15 (1.8)	
Thyroid disease	25 (7.6)	2 (3.3)	14 (9.5)	1 (3.7)	8 (8.7)	38 (4.6)	
Depression	15 (4.6)	2 (3.3)	2 (1.4)	1 (3.7)	10 (10.9)	41 (5.0)	
Degenerative arthritis	100 (30.6)	25 (41.0)	45 (30.6)	5 (18.5)	25 (27.2)	217 (26.2)	
Back pain	77 (23.5)	17 (27.9)	30 (20.4)	3 (11.1)	27 (29.3)	255 (30.8)	
Rheumatoid arthritis	17 (5.2)	3 (4.9)	8 (5.4)	**—**	6 (6.5)	59 (7.1)	
Other	82 (25.1)	16 (26.2)	35 (23.8)	9 (33.3)	22 (23.9)	232 (28.0)	
Time since diagnosis (years), mean ± SD	5.9 ± 2.8	7.8 ± 2.9	5.1 ± 2.3	4.2 ± 2.2	6.3 ± 2.8		**<0.001**
Location primary GIST, *n* (%)							**<0.001**^a^
Stomach	207 (63.1)	46 (75.4)	100 (68.0)	19 (70.4)	42 (45.2)		
Small intestine	79 (24.1)	7 (11.5)	33 (22.4)	8 (29.6)	31 (33.3)		
Rectum	21 (6.4)	3 (4.9)	11 (7.5)	**—**	7 (7.5)		
Other	21 (6.4)	5 (8.2)	3 (2.0)	**—**	13 (14.0)		
Received TKI at some point, *n* (%)	219 (66.8)	14 (23.0)	85 (57.8)	27 (100)	93 (100)		**<0.001**^a^
Neoadjuvant	39 (17.8)	9 (64.3)	30 (35.3)	**—**	**—**		
Adjuvant	45 (20.5)	1 (7.1)	30 (35.3)	14 (51.9)	**—**		
Both neoadjuvant and adjuvant	42 (19.2)	4 (28.6)	25 (29.4)	13 (48.1)	**—**		
Palliative	93 (42.5)	**—**	**—**	**—**	93 (100)		
Last type of TKI received, *n* (%)							
Imatinib	201 (91.8)	14 (100)	84 (98.8)	27 (100)	76 (81.7)		
Sunitinib	9 (4.1)	**—**	1 (1.2)	**—**	8 (8.6)		
Regorafenib	6 (2.7)	**—**	**—**	**—**	6 (6.5)		
Ripretinib	2 (0.9)	**—**	**—**	**—**	2 (2.2)		
Cabozantinib	1 (0.5)	**—**	**—**	**—**	1 (1.1)		
Currently on TKI, *n* (%)	116 (35.4)	**—**	**—**	27 (100)	89 (95.7)^c^		
Had surgery for the GIST at some point, *n* (%)	300 (91.5)	59 (96.7)	146 (99.3)	27 (100.0)	68 (73.1)		**<0.001**^a^

Bold values are statistically significant values.

FU, follow-up; GIST, gastrointestinal stromal tumor; SD, standard deviation; TKI, tyrosine kinase inhibitor.

a Likelihood ratio.

b Low (primary and secondary education), intermediate [(secondary) vocational education], and high (higher vocational education and academic education) educational level.

c Four patients were not currently on a TKI, but their most recent treatment was a TKI with palliative intent; for two patients this was imatinib, for one patient sunitinib, and for one patient cabozantinib.

### HRQoL of patients with GIST compared with a normative population

**Figure 1:**
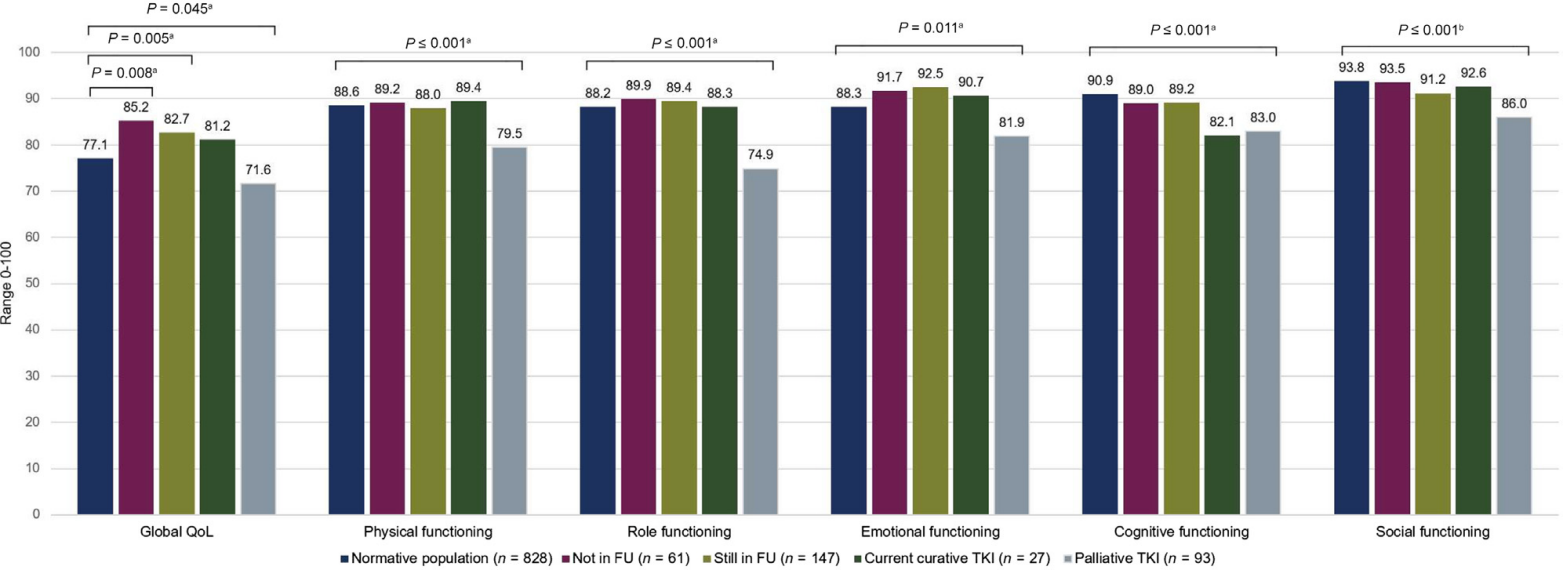
**Comparison of mean scores on global QoL and functioning scales of the EORTC QLQ-C30 between an age- and sex-matched normative population and patients with GIST divided into four subgroups: treatment completed and discharged from FU, treatment completed and in FU, on curative TKI treatment, and palliative treatment—on the global QoL and functioning scales, higher scores indicate a better global quality of life and functioning.** The mean differences between both groups were considered ^a^small and ^b^medium.^21^ EORTC QLQ-C30, European Organization for Research and Treatment of Cancer Quality of Life Core Questionnaire; FU, follow-up; GIST, gastrointestinal stromal tumor; QoL, quality of life; TKI, tyrosine kinase inhibitor.

In total, 828 panel members were matched to 328 patients (ratio 1 : 2.5), with their characteristics presented in Table 1. Patients with GIST that had completed their curative treatment, including those discharged from FU (mean ± SD 85.2 ± 14.0) and those still in FU (mean ± SD 82.7 ± 15.0), scored significantly higher on global QoL compared with the normative population (mean ± SD 77.1 ± 18.2), while patients treated with a palliative intent (mean ± SD 71.6 ± 19.4) scored significantly lower than the normative population (Figure 1; Supplementary Table S2, available at https://doi.org/10.1016/j.esmorw.2024.100037). All differences in mean global QoL scores were considered small.^21^ On all functioning scales, patients that completed treatment and those on curative TKIs had comparable functioning scores to the normative population, except for cognitive functioning, where patients on curative TKI treatment had scores similar to patients on palliative TKI treatment. As expected, patients in the palliative setting scored significantly lower on all functioning scales, with small to medium mean differences, compared with the normative population.

### Disease- and treatment-specific symptoms

**Figure 2:**
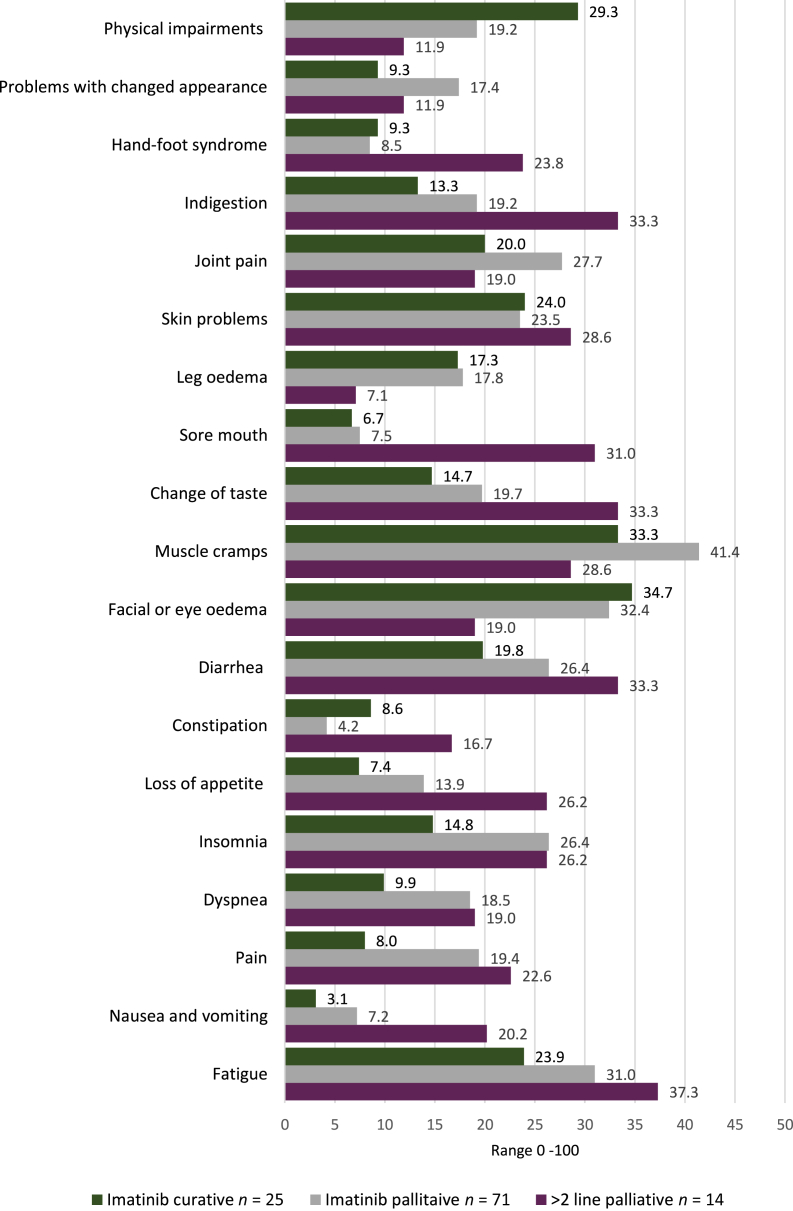
**Mean symptom scores of the symptom scales among patients with GIST on curative imatinib (*n* = 25), palliative imatinib (*n* = 71), and further lines(2 or >)of palliative TKI therapy (*n* = 14).** Scores range from 0 to 100, with higher scores indicating a higher symptom burden. GIST, gastrointestinal stromal tumor; TKI, tyrosine kinase inhibitor.

Well-known side-effects of imatinib, including muscle cramps, leg and facial edema, and fatigue were common in both imatinib-treated groups (Figure 2). Patients on curative imatinib suffered the most from facial edema (34.7), muscle cramps (33.3), and physical impairments (29.3), such as not being able to achieve the same level in sports. By contrast, muscle cramps (41.4), facial edema (32.4), and fatigue (31.0) were most commonly reported in patients on palliative imatinib. Patients on later treatment lines suffered mostly from fatigue (37.3), indigestion (33.3), diarrhea (33.3), change of taste (33.3), and a sore mouth (31.0).

### Factors associated with HRQoL

Results of our multiple linear regression analyses for global QoL and the five functioning scales are presented in Table 2, with the complete analyses in Supplementary Table S3, available at https://doi.org/10.1016/j.esmorw.2024.100037. A worse global QoL score was associated with having two or more comorbidities (*β* = −0.20), fatigue (*β* = −0.31), pain (*β* = −0.34), dyspnea (*β* = −0.10), diarrhea (*β* = −0.10), and financial difficulties (*β* = −0.12). Older age (*β* = −0.13), having two or more comorbidities (*β* = −0.12), fatigue (*β* = −0.30), pain (*β* = −0.29), dyspnea (*β* = −0.31), and financial difficulties (*β* = −0.10) were associated with worse physical functioning, whereas a high socioeconomic status (*β* = 0.10) was associated with better physical functioning. Worse role functioning was associated with fatigue (*β* = −0.36), pain (*β* = −0.33), and dyspnea (*β* = −0.17). Older age (*β* = 0.11) was associated with better emotional functioning, while fatigue (*β* = −0.16), pain (*β* = −0.14), insomnia (*β* = −0.28), and financial difficulties (*β* = −0.15) were associated worse emotional functioning. Fatigue (*β* = −0.43) and financial difficulties (*β* = −0.25) were associated with worse cognitive functioning, and worse social functioning was associated with fatigue (*β* = −0.23), pain (*β* = −0.35), and financial difficulties (*β* = −0.24).

**Table 2:** Standardized betas (β) of multiple linear regression analyses evaluating the association of independent variables with the HRQoL scales

		EORTC QLQ-C30 scales^a^					
		Global QoL	Physical functioning	Role functioning	Emotional functioning	Cognitive functioning	Social functioning
		*R*^2^ = 0.60	*R*^2^ = 0.55	*R*^2^ = 0.52	*R*^2^ = 0.45	*R*^2^ = 0.35	*R*^2^ = 0.48
Sex	Male	Reference	Reference	Reference			Reference
	Female	0.02	−0.06	−0.03	N/A	N/A	0.05
Age		N/A	−0.13^b^	N/A	0.11^c^	N/A	N/A
Socioeconomic status	Low		Reference	Reference			
	High	N/A	0.10^c^	0.07	N/A	N/A	N/A
Educational level	Low/intermediate		Reference				
	High	N/A	0.05	N/A	N/A	—	N/A
Number of comorbidities	None	Reference	Reference	Reference	Reference	Reference	Reference
	1	−0.07	−0.04	N/A	0.05	0.07	−0.004
	≥2	−0.20^b^	−0.12^c^	−0.02	−0.01	0.02	−0.03
Time since diagnosis		N/A	N/A	N/A	−0.08	N/A	N/A
Location	Stomach	Reference	Reference	Reference			
	Other than stomach	−0.01	−0.04	−0.06	N/A	N/A	N/A
Had surgery for the GIST at some point	No	Reference					
	Yes	0.05	N/A	N/A	N/A	N/A	N/A
Current TKI	No	Reference	Reference	Reference	Reference	Reference	Reference
	Yes	0.03	0.01	0.04	0.02	−0.10	0.05
Current treatment setting	Curative	Reference	Reference	Reference	Reference	Reference	Reference
	Palliative	−0.05	−0.04	−0.06	−0.08	0.13	0.08
Fatigue	Range 0-100	−0.31^b^	−0.30^b^	−0.36^b^	−0.16^c^	−0.43^b^	−0.23^b^
Nausea and vomiting	Range 0-100	−0.07	0.05	0.03	−0.08	−0.02	−0.07
Pain	Range 0-100	−0.34^b^	−0.29^b^	−0.33^b^	−0.14^b^	0.01	−0.35^b^
Dyspnea	Range 0-100	−0.10^c^	−0.31^b^	−0.17^b^	0.03	0.06	−0.04
Insomnia	Range 0-100	0.00	0.04	0.06	−0.28^b^	−0.06	−0.09
Loss of appetite	Range 0-100	0.07	0.11^c^	0.02	−0.08	−0.03	−0.10
Constipation	Range 0-100	−0.06	0.03	0.03	−0.05	−0.03	0.09
Diarrhea	Range 0-100	−0.10^c^	0.05	−0.04	−0.05	0.001	0.01
Financial difficulties	Range 0-100	−0.12^b^	−0.10^c^	−0.09	−0.15^b^	−0.25^b^	−0.24^b^

The results of the full multiple linear regression analysis are available as Supplementary Table S3. Here we only report standardized betas of the multivariable logistic regression.

EORTC QLQ-C30, European Organization for Research and Treatment of Cancer Quality of Life Core Questionnaire; GIST, gastrointestinal stromal tumor; HRQoL, Health-related quality of life; TKI, tyrosine kinase inhibitor.

a Higher score indicates better functioning.

b <0.01.

c <0.05.

### Sensitivity analysis

Results of our sensitivity analyses are presented in Supplementary Table S3, available at https://doi.org/10.1016/j.esmorw.2024.100037. Fatigue remained associated with lower scores on global QoL (*β* = −0.42) and most functioning scales (*β* = −0.28 to −0.44), except for emotional functioning. Pain was no longer associated with worse emotional functioning, but still with worse global QoL (*β* = −0.28), physical (*β* = −0.35), role (*β* = −0.35), and social functioning (*β* = −0.41). Dyspnea was associated with worse physical (*β* = −0.31) and role functioning (*β* = −0.21), but not anymore with global QoL. Financial difficulties, which were associated with all scales except for role functioning, only remained associated with worse cognitive (*β* = −0.38) and social functioning (*β* = −0.27).

## Discussion

So far, only a few studies have addressed HRQoL in patients with GIST.^8^ This cross-sectional study was one of the first studies to describe the HRQoL of patients with GIST in different treatment phases and settings, and to make a comparison with an age- and sex-matched normative population. Incorporation of the patient perspective through the use of PROs is becoming more important, both as a component of patient-centered care and as an outcome in research.^13^^,^^22^^,^^23^

In our study, GIST survivors that completed their curative treatment had a significantly better global QoL compared with the normative population. This was in accordance with findings of a study in long-term (>10 years) cancer survivors, where disease-free breast, colorectal, and prostate cancer survivors had a significantly higher global QoL compared with that of controls of the same age.^24^ The fact that patients have survived a potentially life-threatening illness may result in them valuing their lives more.

Studies assessing HRQoL in patients with GIST on adjuvant imatinib are scarce.^25^^,^^26^ One study investigated the tolerability of adjuvant imatinib for 5 years and reported a stable QoL throughout the 60 months of treatment.^25^ Another study evaluating imatinib adherence in the adjuvant setting^26^ reported lower global QoL and social functioning scores, higher role functioning scores, and equal other functioning scores compared with our patients on (neo)adjuvant imatinib. Differences found may be more country specific, possibly related to the way health care and health insurance are organized. In the Netherlands, most patients are treated and receive FU in expertise centers with treatment and health care costs being covered by health insurance. In addition, Dutch patients with GIST had better HRQoL scores than German patients with GIST.^27^ When focusing on global QoL, Dutch patients not or previously on TKI treatment scored on average 17.8 points higher, patients on curative TKI treatment 21.6 points higher, and patients on palliative TKI treatment 8.3 points higher than German patients in the same treatment phase. Although for the last group we have to take into account that 80% of our palliative patients were treated with imatinib, whereas this was only 50% in the German patients. Patients on further lines often report more side-effects (i.e. fatigue or sore mouth) or symptoms (i.e. indigestion), as was also the case in our study, which might explain their lower global QoL. Besides global QoL, Dutch patients with GIST scored on average 17.6-23.5 points higher on role, 23.6-28.1 points higher on emotional, and 22.3-27.9 points higher on social functioning, respectively. The Dutch health care system has similarities with the German system, indicating cultural influences, as was reported earlier by Nolte et al.^28^

In line with our expectations, patients on TKIs with a palliative intent had a significantly lower global QoL compared with the normative population and patients on curative TKI treatment. Although HRQoL is the most affected in the palliative treatment setting, imatinib, sunitinib, and regorafenib were all approved for the treatment of advanced GIST based on studies without HRQoL data,^29^, ^30^, ^31^ highlighting the value of including PROs for evaluating treatments in this group. The HRQoL scores of patients with advanced GIST on imatinib in a Malaysian clinic were comparable with our patients on palliative TKIs.^32^ Compared with other patients with metastatic cancer, patients with GIST scored 13.2-15.3 points higher on global QoL, role, and emotional and social functioning, while physical and cognitive functioning scores were similar.^33^

Fatigue, a common side-effect of imatinib and further lines of TKI treatment,^8^^,^^34^ is a key symptom when it comes to HRQoL, as it was associated with lower scores on all HRQoL scales. This is supported by a previous study,^27^ where severely fatigued patients with GIST reported significantly lower HRQoL scores than nonseverely fatigued patients.^35^ In that study, one in three patients suffered from severe fatigue, and fatigue severity was associated with TKI use, underlining the relevance of this symptom, also considering that patients with advanced GIST depend on long-term TKI treatment. Therefore it is important for clinicians to address fatigue, as patients can benefit from appropriate management. Both internet-delivered cognitive behavior therapy and online psychoeducation were shown to be effective in reducing fatigue and improving the HRQoL of patients with GIST.^36^ Besides symptoms, financial difficulties, though uncommon among Dutch patients with GIST (11.7%),^37^ were associated with reduced scores on almost all HRQoL scales, except for role functioning.

A notable finding was the lower scores on cognitive functioning in patients treated with TKIs in the curative as well as the palliative setting, suggesting that TKIs, in our study mainly imatinib, might influence cognitive function in terms of concentration and memory. Of the registered TKIs in GIST treatment, avapritinib is known for its cognitive side-effects.^38^ Although cognitive impairment has been reported in sunitinib- as well as imatinib-treated patients,^27^^,^^39^^,^^40^ it is not commonly recognized by clinicians.^41^ That cognitive impairment is more common than thought was highlighted by a survey study, where almost two out of the three GIST survivors self-reported cognitive impairment with a significant negative impact on their QoL.^42^

In the available literature, HRQoL in patients treated with imatinib, regorafenib, and ripretinib remained stable, whereas sunitinib-treated patients reported a decreased HRQoL.^8^ Because of the low number of patients on further lines of TKIs, we were not able to evaluate the effect of these treatments on HRQoL. In addition, the cross-sectional design limited us to study causalities and changes in HRQoL over time. Furthermore, this was a multicenter study conducted only in the Netherlands, which could impede the generalizability as the Dutch health care and health insurance differ from other countries. Another limitation of our study is that there could be some selection bias, as reasons for not participating were not collected, and could be due to either poor health or the absence of symptoms.

Strengths of our study were the use of PRO measures, which are not widely used in GIST research. The relatively high response rate (63%) in our study underlines the willingness of patients with GIST to participate in this type of study. Interestingly, our nonresponder analysis (Supplementary Table S1, available at https://doi.org/10.1016/j.esmorw.2024.100037) showed that more men than women participated in our study. In line with previous studies,^43^^,^^44^ nonresponders more often had a lower socioeconomic status than responders. In future studies, we should try to find ways to motivate these patients to participate as well.^45^ Furthermore, we should take into account the improved overall survival of patients with GIST over the past decades, making it more important to not only assess treatment effectiveness in terms of objective clinical outcomes (e.g. radiological response and survival), but also in terms of PROs. Lastly, our study represented the largest cohort of patients with GIST in which HRQoL was examined. Our diverse study population, including patients in different treatment phases and settings, allowed us to compare the HRQoL of these different groups, rather than focusing on HRQoL in patients with advanced GIST on specific TKI treatments.

### Conclusion

The HRQoL of patients who have completed their curative treatment or are treated with (neo)adjuvant imatinib was better or comparable with the normative population, while patients with GIST on palliative TKI treatment had a worse HRQoL. Fatigue had the greatest impact on HRQoL, in addition to pain, dyspnea, having two or more comorbidities, and financial difficulties. With these results, medical oncologists can reassure patients with GIST treated in an adjuvant setting that their HRQoL will not be permanently affected by imatinib, and provide appropriate support to patients in the palliative setting, who benefit from an increase in lifetime duration from TKIs, but also need to deal with a life in which their HRQoL is affected by the disease and treatment.
