# Supplementary material for: Health-related quality of life in patients with gastrointestinal stromal tumor: data from a real-world cohort compared with a normative population
Source: ESMO Real World Data Digit Oncol. 2024 Apr 24;4:100037. doi: 10.1016/j.esmorw.2024.100037 (PMC12836709; doi:10.1016/j.esmorw.2024.100037)
Supplement: Supplementary Tables [file mmc1.docx]

Supplementary table A. Characteristics of responders and non-responders

|  | **Responders**  **(n=328)** | **Non-responders**  **(n=193)** | ***p*-value** |
| --- | --- | --- | --- |
| Sex *n (%)*  Male  Female | 174 (53.0)  154 (47.0) | 88 (45.6)  105 (54.4) | .100 |
| Socio-economic status *n (%)*  Low  Intermediate  High | 90 (27.4)  132 (40.2)  106 (32.3) | 68 (35.2)  57 (29.5)  68 (35.2) | **.037** |
| Age at diagnosis *Mean ± SD* | 60.8 ± 10.6 | 60.8 ± 13.4 | .981 |
| Time since diagnosis in years *Mean ± SD* | 5.9 ± 2.8 | 5.3 ± 2.8 | **.014** |
| Location primary GIST *n (%)*  Stomach or peritoneum  Other | 210 (64.0)  118 (36.0) | 124 (64.2)  69 (35.8) | .745 |
| Surgery at some point* | 287 (87.5) | 164 (85.0) | .414 |
| Received TKI at some point* | 214 (65.2) | 114 (59.1) | .261 |
| *Data in this table is solely based on NCR data and differs from that in the manuscript | | | |

Supplementary table B. Comparison of mean scores on global QoL, functioning and symptom scales of the EORTC QLQ-C30 among GIST patients in different FU and treatment settings and an age- and sex-matched norm population

| **Scale** | **Group** | **Mean** | **SD** | **Mean difference** | **Posthoc Bonferroni**  ***p*-value** |
| --- | --- | --- | --- | --- | --- |
| Global QoL | Norm population  Not in FU  Still in FU  Curative TKI  Palliative TKI | 77.1  85.2  82.7  81.2  71.6 | 18.2  14.0  15.0  12.6  19.4 | -8.1*  -5.6*  -4.0*  5.5* | **.008**  **.005**  1.000  **.045** |
| Physical functioning | Norm population  Not in FU  Still in FU  Curative TKI  Palliative TKI | 88.6  89.2  88.0  89.4  79.5 | 16.0  18.7  14.7  15.8  20.3 | -0.6  0.7  -0.7  9.2* | 1.000  1.000  1.000  **<.001** |
| Role functioning | Norm population  Not in FU  Still in FU  Curative TKI  Palliative TKI | 88.2  89.9  89.4  88.3  74.9 | 20.7  19.8  19.7  23.0  26.8 | -1.6  -1.1  -0.1  13.3* | 1.000  1.000  1.000  **<.001** |
| Emotional functioning | Norm population  Not in FU  Still in FU  Curative TKI  Palliative TKI | 88.3  91.7  92.5  90.7  81.9 | 17.9  15.3  13.7  15.4  19.4 | -3.4  -4.2  -2.4  6.3* | 1.000  .072  1.000  **.011** |
| Cognitive functioning | Norm population  Not in FU  Still in FU  Curative TKI  Palliative TKI | 90.9  89.0  89.2  82.1  83.0 | 15.6  17.8  15.8  25.3  18.5 | 1.9  1.7  8.8*  7.9* | 1.000  1.000  .056  **<.001** |
| Social functioning | Norm population  Not in FU  Still in FU  Curative TKI  Palliative TKI | 93.8  93.5  91.2  92.6  86.0 | 14.8  14.1  19.5  13.3  23.3 | 0.3  2.6  1.2  7.8* | 1.000  .813  1.000  **<.001** |
| Fatigue | Norm population  Not in FU  Still in FU  Curative TKI  Palliative TKI | 16.8  14.9  16.0  23.9  31.3 | 20.5  18.6  18.7  25.4  23.1 | 2.0  0.9  -7.0*  -14.5** | 1.000  1.000  .810  **<.001** |
| Nausea and vomiting | Norm population  Not in FU  Still in FU  Curative TKI  Palliative TKI | 2.8  5.4  3.4  3.1  9.2 | 11.3  17.1  11.1  6.6  18.6 | -2.5  -0.5  -0.3  -6.3* | 1.000  1.000  1.000  **<.001** |
| Pain | Norm population  Not in FU  Still in FU  Curative TKI  Palliative TKI | 14.6  9.2  7.9  8.0  19.7 | 22.5  16.5  17.4  16.9  25.6 | 5.4  6.8*  6.6*  -5.0 | .732  **.006**  1.000  .392 |
| Dyspnoea | Norm population  Not in FU  Still in FU  Curative TKI  Palliative TKI | 7.7  13.1  9.0  9.9  18.0 | 17.5  23.5  17.7  20.3  24.1 | -5.4*  -1.3  -2.2  -10.3** | .344  1.000  1.000  **<.001** |
| Insomnia | Norm population  Not in FU  Still in FU  Curative TKI  Palliative TKI | 18.0  12.5  14.9  14.8  26.6 | 26.1  24.3  25.5  23.3  28.5 | 5.5*  3.1  3.2  -8.6* | 1.000  1.000  1.000  **.033** |
| Loss of appetite | Norm population  Not in FU  Still in FU  Curative TKI  Palliative TKI | 3.8  6.5  3.7  7.4  15.4 | 13.7  18.4  11.2  19.2  26.6 | -2.8  0.1  -3.6  -11.6* | 1.000  1.000  1.000  **<.001** |
| Constipation | Norm population  Not in FU  Still in FU  Curative TKI  Palliative TKI | 4.9  3.6  6.0  8.6  6.0 | 14.4  13.7  15.6  17.5  16.3 | 1.3  -1.1  -3.8  -1.1 | 1.000  1.000  1.000  1.000 |
| Diarrhoea | Norm population  Not in FU  Still in FU  Curative TKI  Palliative TKI | 4.1  8.3  5.3  19.8  26.6 | 13.2  18.3  14.0  31.0  31.1 | -4.2  -1.2  -15.6**  -22.4** | .624  1.000  **<.001**  **<.001** |
| Financial difficulties | Norm population  Not in FU  Still in FU  Curative TKI  Palliative TKI | 3.5  1.8  3.7  3.7  9.7 | 12.9  9.9  15.3  10.7  25.2 | 1.7  -0.2  -0.2  -6.3* | 1.000  1.000  1.000  **<.001** |
| *On global QoL and functioning scales, higher scores indicate a better global quality of life and functioning, while on the symptom scales higher scores indicate a higher symptom burden - The mean differences between both groups were considered *small, **medium or ***large [21]* | | | | | |

Supplementary table C. Multiple linear regression analyses for the total GIST population and for GIST patients on current TKI treatment (sensitivity analysis) evaluating the association of independent variables with the outcomes global QoL, physical, role, emotional, cognitive and social functioning

| Global QoL | | Total GIST population (n=319) - R² = 0.60 | | | Patients on current TKI treatment (n=114) - R² = 0.72 | | |
| --- | --- | --- | --- | --- | --- | --- | --- |
|  |  | **B (95% CI)** | **β** | ***p-*value** | **B (95% CI)** | **β** | ***p-*value** |
| Sex | Male  Female | Reference  0.75 (-1.87 – 3.37) | 0.02 | .575 | Reference  -5.33 (-9.80 – -0.85) | -0.14 | **.020** |
| Number of comorbidities | None  1  >2 | Reference  -3.02 (-6.43 – 0.40)  -6.59 (-9.57 – -3.61) | -0.07  -0.20 | .083  **<.001** | Reference  -3.29 (-9.24 – 2.65)  -11.62 (-16.46 – -6.78) | -0.07  -0.31 | .274  **<.001** |
| Location | Stomach  Other than stomach | Reference  -0.29 (-2.94 – 2.36) | -0.01 | .828 | Reference  -3.61 (-7.88 – 0.66) | -0.10 | .096 |
| Had surgery for the GIST at some point | No  Yes | Reference  2.73 (-2.19 – 7.64) | 0.05 | .276 | Reference  3.48 (-1.87 – 8.82) | 0.08 | .200 |
| Current TKI | No  Yes | Reference  0.97 (-3.36 – 5.29) | 0.03 | .661 |  |  |  |
| Current treatment setting | Curative  Palliative | Reference  -1.98 (-6.79 – 2.83) | -0.05 | .419 | Reference  -2.80 (-8.11 – 2.52) | -0.06 | .299 |
| Fatigue | Range 0 - 100 | -0.24 (-0.32 – -0.16)* | -0.31 | **<.001** | -0.33 (-0.44 – -0.22)* | -0.42 | **<.001** |
| Nausea and vomiting | Range 0 - 100 | -0.08 (-0.18 – 0.03) | -0.07 | .142 | -0.06 (-0.20 – 0.08) | -0.06 | .382 |
| Pain | Range 0 - 100 | -0.28 (-0.35 – -0.21)* | -0.34 | **<.001** | -0.21 (-0.32 – -0.11)* | -0.28 | **<.001** |
| Dyspnoea | Range 0 - 100 | -0.08 (-0.14 – -0.01) | -0.10 | **.020** | -0.07 (-0.17 – 0.02) | -0.09 | .115 |
| Insomnia | Range 0 - 100 | 0.002 (-0.05 – 0.06) | 0.002 | .954 | 0.09 (0.003 – 0.18) | 0.14 | **.043** |
| Loss of appetite | Range 0 - 100 | 0.06 (-0.02 – 0.14) | 0.07 | .144 | 0.06 (-0.04 – 0.15) | 0.08 | .224 |
| Constipation | Range 0 - 100 | -0.06 (-0.14 – 0.02) | -0.06 | .149 | -0.26 (-0.40 – -0.11)* | -0.23 | **<.001** |
| Diarrhoea | Range 0 - 100 | -0.06 (-0.12 – -0.004) | -0.10 | **.037** | -0.05 (-0.12 – 0.02) | -0.08 | .170 |
| Financial difficulties | Range 0 - 100 | -0.11 (-0.19 – -0.04)* | -0.12 | **.004** | 0.004 (-0.10 – 0.11) | 0.01 | .940 |
| Physical functioning | | **Total GIST population (n=318)** - R² = 0.55 | | | **Patients on current TKI treatment (n=113) -** R² = 0.61 | | |
|  |  | **B (95% CI)** | **β** | ***p-*value** | **B (95% CI)** | **β** | ***p-*value** |
| Sex | Male  Female | Reference  -1.97 (-4.95 – 1.00) | -0.06 | .192 | Reference  -4.35 (-10.18 – 1.47) | -0.11 | .141 |
| Age |  | -0.23 (-0.38 – -0.09) | -0.13 | **.002** | -0.38 (-0.68 – -0.07) | -0.18 | **.018** |
| Socio-economic status | Low  High | Reference  3.47 (0.61 – 6.33) | 0.10 | **.018** | Reference  4.60 (-0.91 – 10.12) | 0.12 | .101 |
| Educational level | Low/intermediate  High | Reference  1.95 (-0.98 – 4.89) | 0.05 | .191 | Reference  0.61 (-4.87 – 6.08) | 0.02 | .827 |
| Number of comorbidities | None  1  >2 | Reference  -1.54 (-5.49 – 2.40)  -4.32 (-7.76 – -0.88) | -0.04  -0.12 | .442  **.014** | Reference  0.54 (-7.24 – 8.31)  -5.50 (-11.93 – 0.92) | 0.01  -0.14 | .891  .092 |
| Location | Stomach  Other than stomach | Reference  -1.45 (-4.48 – 1.58) | -0.04 | .347 | Reference  -5.00 (-10.45 – 0.54) | -0.13 | .076 |
| Current TKI | No  Yes | Reference  0.24 (-4.72 – 5.19) | 0.01 | .926 |  |  |  |
| Current treatment setting | Curative  Palliative | Reference  -1.68 (-7.00 – 3.64) | -0.04 | .535 | Reference  -1.01 (-7.76 – 5.73) | -0.02 | .766 |
| Fatigue | Range 0 - 100 | -0.24 (-0.34 – -0.15)* | -0.30 | **<.001** | -0.30 (-0.45 – -0.15)* | -0.36 | **<.001** |
| Nausea and vomiting | Range 0 - 100 | 0.07 (-0.05 – 0.18) | 0.05 | .272 | -0.002 (-0.18 – 0.18) | -0.002 | .985 |
| Pain | Range 0 - 100 | -0.25 (-0.33 – -0.17)* | -0.29 | **<.001** | -0.28 (-0.42 – -0.15)* | -0.35 | **<.001** |
| Dyspnoea | Range 0 - 100 | -0.26 (-0.33 – -0.18)* | -0.31 | **<.001** | -0.26 (-0.38 – -0.14)* | -0.31 | **<.001** |
| Insomnia | Range 0 - 100 | 0.03 (-0.03 – 0.09) | 0.04 | .339 | 0.10 (-0.02 – 0.21)* | 0.14 | .102 |
| Loss of appetite | Range 0 - 100 | 0.10 (0.01 – 0.19) | 0.11 | **.033** | 0.10 (-0.02 – 0.23) | 0.13 | .100 |
| Constipation | Range 0 - 100 | 0.03 (-0.06 – 0.13) | 0.03 | .473 | 0.07 (-0.12 – 0.25) | 0.06 | .467 |
| Diarrhoea | Range 0 - 100 | 0.04 (-0.03 – 0.10) | 0.05 | .290 | 0.06 (-0.03 – 0.15) | 0.10 | .183 |
| Financial difficulties | Range 0 - 100 | -0.10 (-0.18 – -0.01)* | -0.10 | **.031** | -0.01 (-0.15 – 0.13) | -0.01 | .919 |
| Role Functioning | | **Total GIST population (n=316)** - R² = 0.52 | | | **Patients on current TKI treatment (n=113) -** R² = 0.62 | | |
|  |  | **B (95% CI)** | **β** | ***p-*value** | **B (95% CI)** | **β** | ***p-*value** |
| Sex | Male  Female | Reference  -1.43 (-5.33 – 2.47) | -0.03 | .470 | Reference  -3.68 (-11.16 – 3.80) | -0.07 | .332 |
| Socio-economic status | Low  High | Reference  3.18 (-0.58 – 6.96) | 0.07 | .097 | Reference  4.01 (-3.05 – 11.08) | 0.08 | .262 |
| Number of comorbidities | None  >2 | Reference  -0.84 (-4.81 – 3.14) | -0.02 | .680 | Reference  -1.82 (-9.02 – 5.37) | -0.03 | .617 |
| Location | Stomach  Other than stomach | Reference  -2.99 (-6.96 – 0.99) | -0.06 | .140 | Reference  -8.70 (-15.76 – -1.65) | -0.16 | **.016** |
| Current TKI | No  Yes | Reference  2.01 (-4.48 – 8.49) | 0.04 | .543 |  |  |  |
| Current treatment setting | Curative  Palliative | Reference  -3.25 (-10.23 – 3.74) | -0.06 | .361 | Reference  -2.28 (-10.75 – 6.20) | -0.04 | .595 |
| Fatigue | Range 0 - 100 | -0.38 (-0.51 – -0.26)* | -0.36 | **<.001** | -0.50 (-0.68 – -0.30)** | -0.44 | **<.001** |
| Nausea and vomiting | Range 0 - 100 | 0.05 (-0.10 – 0.20) | 0.03 | .526 | 0.11 (-0.12 – 0.35) | 0.08 | .322 |
| Pain | Range 0 - 100 | -0.38 (-0.49 – -0.27)* | -0.33 | **<.001** | -0.38 (-0.55 – -0.20)* | -0.35 | **<.001** |
| Dyspnoea | Range 0 - 100 | -0.18 (-0.28 – -0.09)* | -0.17 | **<.001** | -0.24 (-0.40 – -0.09)* | -0.21 | **.002** |
| Insomnia | Range 0 - 100 | 0.05 (-0.03 – 0.13) | 0.06 | .243 | -0.01 (-0.16 – 0.14) | -0.01 | .937 |
| Loss of appetite | Range 0 - 100 | 0.03 (0.10 – 0.15) | 0.02 | .670 | 0.06 (-0.10 – 0.22) | 0.06 | .443 |
| Constipation | Range 0 - 100 | 0.04 (-0.08 – 0.17) | 0.03 | .494 | -0.01 (-0.25 – 0.23) | -0.01 | .928 |
| Diarrhoea | Range 0 - 100 | -0.04 (-0.13 – 0.05) | -0.04 | .363 | 0.003 (-0.12 – 0.12) | 0.003 | .954 |
| Financial difficulties | Range 0 - 100 | -0.11 (-0.23 – 0.002)* | -0.09 | .053 | 0.09 (-0.09 – 0.27) | 0.08 | .311 |
| Emotional functioning | | **Total GIST population (n=316)** - R² = 0.45 | | | **Patients on current TKI treatment (n=113) -** R² = 0.45 | | |
|  |  | **B (95% CI)** | **β** | ***p-*value** | **B (95% CI)** | **β** | ***p-*value** |
| Age |  | 0.17 (0.03 – 0.32) | 0.11 | **.020** | 0.32 (-0.02 – 0.66) | 0.16 | .066 |
| Number of comorbidities | None  1  >2 | Reference  2.09 (-1.84 – 6.03)  -0.16 (-3.62 – 3.31) | 0.05  -0.01 | .296  .930 | Reference  2.45 (-5.93 – 10.83)  -0.93 (-8.07 – 6.21) | 0.05  -0.03 | .563  .797 |
| Time since diagnosis |  | -0.49 (-1.01 – 0.03) | -0.08 | .066 | -0.11 (-1.23 – 1.01) | -0.02 | .844 |
| Current TKI | No  Yes | Reference  0.83 (-4.16 -5.81) | 0.02 | .745 |  |  |  |
| Current treatment setting | Curative  Palliative | Reference  -2.87 (-8.19 – 2.46) | -0.08 | .290 | Reference  -5.56 (-13.09 – 2.00) | -0.13 | .147 |
| Fatigue | Range 0 - 100 | -0.12 (-0.21 – -0.02) | -0.16 | **.014** | -0.15 (-0.32 – 0.01) | -0.19 | .073 |
| Nausea and vomiting | Range 0 - 100 | -0.09 (-0.21 – 0.02) | -0.08 | .110 | 0.10 (-0.10 – 0.29) | 0.09 | .320 |
| Pain | Range 0 - 100 | -0.12 (-0.20 – -0.03) | -0.14 | **.006** | -0.14 (-0.28 – 0.004) | -0.18 | .056 |
| Dyspnoea | Range 0 - 100 | 0.02 (-0.06 – 0.10) | 0.03 | .582 | -0.03 (-0.16 – 0.11) | -0.03 | .693 |
| Insomnia | Range 0 - 100 | -0.18 (-0.24 – -0.12)* | -0.28 | **<.001** | -0.06 (-0.18 – 0.07) | -0.08 | .376 |
| Loss of appetite | Range 0 - 100 | -0.07 (-0.16 – 0.03) | -0.08 | .169 | -0.06 (-0.20 – 0.08) | -0.08 | .393 |
| Constipation | Range 0 - 100 | -0.05 (-0.14 – 0.05) | -0.05 | .329 | -0.25 (-0.45 – -0.05)* | -0.22 | **.015** |
| Diarrhoea | Range 0 - 100 | -0.03 (-0.10 – 0.03) | -0.05 | .315 | -0.07 (-0.17 – 0.03) | -0.12 | .162 |
| Financial difficulties | Range 0 - 100 | -0.14 (-0.22 – -0.05)* | -0.15 | **.002** | -0.11 (-0.26 – 0.05)* | -0.13 | .169 |
| Cognitive functioning | | **Total GIST population (n=316)** - R² = 0.35 | | | **Patients on current TKI treatment (n=113) -** R² = 0.42 | | |
|  |  | **B (95% CI)** | **β** | ***p-*value** | **B (95% CI)** | **β** | ***p-*value** |
| Number of comorbidities | None  1  >2 | Reference  2.88 (-1.75 – 7.51)  0.85 (-3.20 – 4.89) | 0.07  0.02 | .222  .680 | Reference  0.21 (-8.80 – 9.22)  2.37 (-5.06 – 9.80) | 0.004  0.06 | .963  .528 |
| Current TKI | No  Yes | Reference  -3.71 (-9.61 – 2.19) | -0.10 | .217 |  |  |  |
| Current treatment setting | Curative  Palliative | Reference  5.00 (-1.25 – 11.25) | 0.13 | .117 | Reference  4.56 (-3.03 – 12.15) | 0.10 | .236 |
| Fatigue | Range 0 - 100 | -0.36 (-0.47 – -0.25)* | -0.43 | **<.001** | -0.37 (-0.54 – -0.20)* | -0.43 | **<.001** |
| Nausea and vomiting | Range 0 - 100 | -0.02 (-0.16 – 0.12) | -0.02 | .796 | -0.04 (-0.25 – 0.18) | -0.03 | .735 |
| Pain | Range 0 - 100 | 0.01 (-0.09 – 0.11) | 0.01 | .834 | 0.02 (-0.12 – 0.17) | 0.02 | .795 |
| Dyspnoea | Range 0 - 100 | 0.05 (-0.04 – 0.14) | 0.06 | .268 | 0.05 (-0.09 – 0.20) | 0.06 | .463 |
| Insomnia | Range 0 - 100 | -0.04 (-0.11 – 0.03) | -0.06 | .302 | -0.01 (-0.14 – 0.13) | -0.01 | .909 |
| Loss of appetite | Range 0 - 100 | -0.03 (-0.14 – 0.08) | -0.03 | .641 | -0.02 (-0.16 – 0.13) | -0.02 | .817 |
| Constipation | Range 0 - 100 | -0.04 (-0.15 – 0.08) | -0.03 | .517 | -0.04 (-0.26 – 0.17) | -0.04 | .697 |
| Diarrhoea | Range 0 - 100 | 0.001 (-0.08 – 0.08) | 0.001 | .990 | 0.05 (-0.06 – 0.16) | 0.08 | .357 |
| Financial difficulties | Range 0 - 100 | -0.25 (-0.35 – -0.15)* | -0.25 | **<.001** | -0.34 (-0.50 – -0.17)* | -0.38 | **<.001** |
| Social functioning | | **Total GIST population (n=316)** - R² = 0.48 | | | **Patients on current TKI treatment (n=113) -** R² = 0.53 | | |
|  |  | **B (95% CI)** | **β** | ***p-*value** | **B (95% CI)** | **β** | ***p-*value** |
| Sex | Male  Female | Reference  2.06 (-1.38 – 5.49) | 0.05 | .239 | Reference  3.09 (-3.58 – 9.76) | 0.07 | .360 |
| Number of comorbidities | None  1  >2 | Reference  -0.17 (-4.67 – 4.33)  -1.04 (-4.97 – 2.89) | -0.004  -0.03 | .940  .603 | Reference  -2.33 (-11.18 – 6.51)  -3.61 (-10.88 – 3.66) | -0.04  -0.08 | .602  .326 |
| Current TKI | No  Yes | Reference  2.19 (-3.52 – 7.90) | 0.05 | .451 |  |  |  |
| Current treatment setting | Curative  Palliative | Reference  3.49 (-2.57 – 9.56) | 0.08 | .258 | Reference  2.01 (-5.50 – 9.51) | 0.04 | .597 |
| Fatigue | Range 0 - 100 | -0.21 (-0.32 – -0.10)* | -0.23 | **<.001** | -0.26 (-0.42 – -0.09)* | -0.28 | **.003** |
| Nausea and vomiting | Range 0 - 100 | -0.09 (-0.23 – 0.04) | -0.07 | .181 | -0.02 (-0.23 – 0.19) | -0.01 | .869 |
| Pain | Range 0 - 100 | -0.33 (-0.43 – -0.24) | -0.35 | **<.001** | -0.37 (-0.52 – -0.21) | -0.41 | **<.001** |
| Dyspnoea | Range 0 - 100 | -0.03 (-0.12 – 0.05) | -0.04 | .441 | -0.01 (-0.15 – 0.13) | -0.01 | .872 |
| Insomnia | Range 0 - 100 | -0.07 (-0.14 – 0.01) | -0.09 | .068 | 0.02 (-0.11 – 0.16) | 0.03 | .750 |
| Loss of appetite | Range 0 - 100 | -0.10 (-0.21 – 0.01) | -0.10 | .063 | -0.08 (-0.22 – 0.07) | -0.09 | .298 |
| Constipation | Range 0 - 100 | 0.11 (-0.002 – 0.22) | 0.09 | .053 | 0.07 (-0.14 – 0.28) | 0.05 | .532 |
| Diarrhoea | Range 0 - 100 | 0.01 (-0.07 – 0.08) | 0.01 | .902 | 0.02 (-0.09 – 0.13) | 0.03 | .721 |
| Financial difficulties | Range 0 - 100 | -0.26 (-0.36 – -0.16)* | -0.24 | **<.001** | -0.26 (-0.42 – -0.10)* | -0.27 | **.002** |
| *The effect size were considered *small, **medium or ***large [21]* | | | | | | | |
